# Supplementary material for: Early absolute lymphocyte count was associated with one-year mortality in critically ill surgical patients: A propensity score-matching and weighting study
Source: PLoS One. 2024 May 30;19(5):e0304627. doi: 10.1371/journal.pone.0304627 (PMC11139264; doi:10.1371/journal.pone.0304627)
Supplement: S1 Fig — Abbreviations: PSM, propensity score matching; IPTW, inverse probability of treatment weighting; CBPS, covariate balancing propensity score; APACHE, acute physiology and chronic health evaluation; CCI, Charlson comorbidity index; RRT, renal replacement therapy. (PDF) [file pone.0304627.s001.pdf]

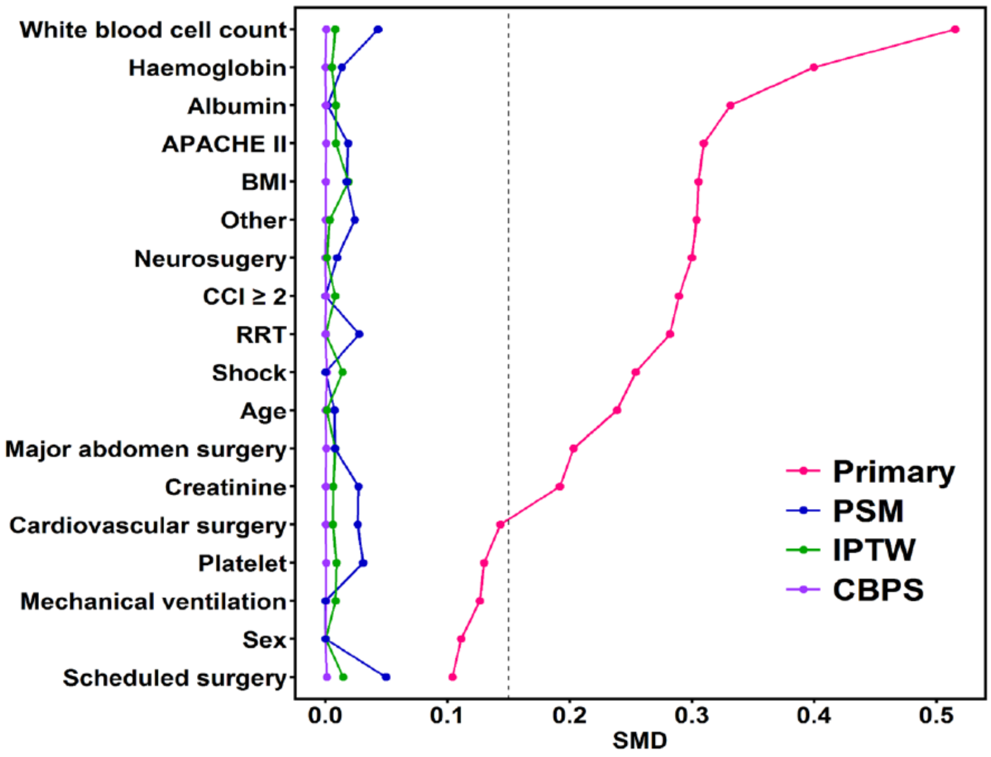

Supplement Figure 1. Standardized mean differences of variables in different patient populations. Abbreviations: PSM, propensity score matching; IPTW, inverse probability of treatment weighting; CBPS, covariate balancing propensity score; APACHE, acute physiology and chronic health evaluation; CCI, Charlson comorbidity index; RRT, renal replacement therapy.
