# Supplementary material for: Early absolute lymphocyte count was associated with one-year mortality in critically ill surgical patients: A propensity score-matching and weighting study
Source: PLoS One. 2024 May 30;19(5):e0304627. doi: 10.1371/journal.pone.0304627 (PMC11139264; doi:10.1371/journal.pone.0304627)
Supplement: S2 Fig — (A) Original population (B) Propensity-score matched population. (PDF) [file pone.0304627.s002.pdf]

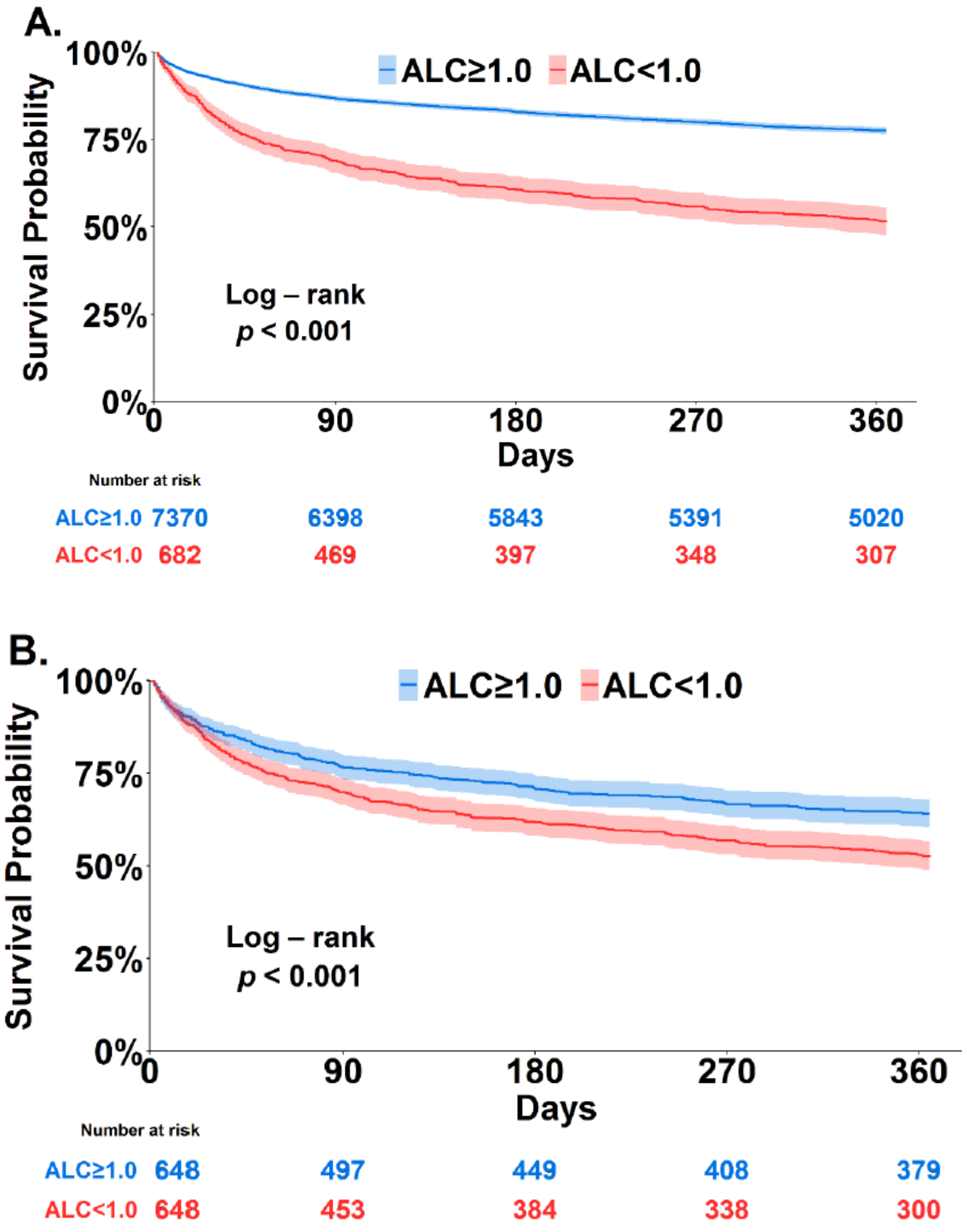

Supplemental Figure 2. Kaplan-Meier survival curves of critically ill surgical patients whose absolute lymphocyte count lower and higher/equal than 1.0 (10^3 /μL) (A) Original population (B) Propensity-score matched population.
