# Supplementary material for: Early absolute lymphocyte count was associated with one-year mortality in critically ill surgical patients: A propensity score-matching and weighting study
Source: PLoS One. 2024 May 30;19(5):e0304627. doi: 10.1371/journal.pone.0304627 (PMC11139264; doi:10.1371/journal.pone.0304627)
Supplement: S1 Table — (PDF) [file pone.0304627.s003.pdf]

Supplement Table 1. Characteristics between the patients categorized by week-1 ALC in the primary cohort and propensity score matched cohort

	Before PSM			After 1:1 PSM		
	ALC≥1000 n=7,370	ALC<1000 n=682	SMD	ALC≥1000 n=648	ALC<1000 n=648	SMD
**Demographic and comorbidity**						
Age, years	60.64 ± 16.09	64.34 ± 14.87	0.239	64.87 ± 15.41	64.3 ± 14.86	0.038
Sex (male)	4581 (62.2%)	460 (67.4%)	0.111	438 (67.6%)	438 (67.6%)	<0.001
Body mass index, kg/m2	24.32 ± 4.52	22.94 ± 4.57	0.305	23.07 ± 4.17	22.97 ± 4.61	0.022
Charlson Comorbidity Index≧2	3214 (43.6%)	395 (57.9%)	0.289	376 (58%)	376 (58%)	<0.001
**Surgical divisions**						
Cardiovascular surgical division	1455 (19.7%)	98 (14.4%)	0.143	95 (14.7%)	97 (15%)	0.009
Neurosurgical division	3764 (51.1%)	248 (36.4%)	0.300	235 (36.3%)	246 (38%)	0.035
General-colorectal surgery divisions	921 (12.5%)	136 (19.9%)	0.203	133 (20.5%)	130 (20.1%)	0.012
Other surgical divisions	1230 (16.7%)	200 (29.3%)	0.304	185 (28.5%)	175 (27%)	0.034
**Scheduled surgery**	4035 (54.7%)	338 (49.6%)	0.104	323 (49.8%)	323 (49.8%)	<0.001
**Severity and managements**						
APACHE II score	20.06 ± 6.89	22.23 ± 7.13	0.310	21.98 ± 6.87	22.01 ± 7.07	0.004
Presence of shock	766 (10.4%)	132 (19.4%)	0.254	116 (17.9%)	116 (17.9%)	<0.001
Receiving mechanical ventilation	5048 (68.5%)	506 (74.2%)	0.126	486 (75%)	486 (75%)	<0.001
Receiving RRT	423 (5.7%)	96 (14.1%)	0.282	89 (13.7%)	88 (13.6%)	0.004
**Laboratory data**						
White blood cell count (10^3/μL)	14.63 ± 3.4	12.72 ± 4	0.515	13.09 ± 3.47	12.97 ± 3.9	0.032
Haemoglobin (g/dL)	13.53 ± 1.57	12.92 ± 1.49	0.400	12.86 ± 1.38	12.96 ± 1.49	0.071
Platelet (10^3/μL)	291.06 ± 58.52	283.52 ± 57.6	0.130	283.23 ± 61.53	285.16 ± 57.68	0.032
Albumin (g/dL)	3.67 ± 0.54	3.5 ± 0.5	0.331	3.53 ± 0.51	3.52 ± 0.51	0.028
Creatinine (mg/dL)	1.09 ± 0.39	1.17 ± 0.44	0.192	1.17 ± 0.43	1.16 ± 0.44	0.034

Abbreviations: ALC, absolute lymphocyte count; SMD, standard mean difference; PSM, propensity score matched; APACHE, acute physiology and chronic health evaluation; RRT, renal replacement therapy.
