# Supplementary material for: Early absolute lymphocyte count was associated with one-year mortality in critically ill surgical patients: A propensity score-matching and weighting study
Source: PLoS One. 2024 May 30;19(5):e0304627. doi: 10.1371/journal.pone.0304627 (PMC11139264; doi:10.1371/journal.pone.0304627)
Supplement: S2 Table — (PDF) [file pone.0304627.s004.pdf]

Supplemental Table 2. Cox proportional hazard regression analysis for the association between the first absolute lymphocyte count and long-term mortality in the critically ill surgical patients

	Univariable Analysis		Multivariable Analysis	
	**HR (95% CI)**	**p-value**	**HR (95% CI)**	**p-value**
**Demographic and comorbidity**				
Age, years	1.027 (1.024-1.030)	<0.001	1.009 (1.006-1.013)	<0.001
Sex (male)	1.289 (1.172-1.418)	<0.001	1.150 (1.041-1.270)	0.006
Body mass index (per 1 kg/m2 increment)	0.949 (0.938-0.959)	<0.001	0.962 (0.952-0.972)	<0.001
CCI ≥ 2	2.665 (2.427-2.927)	<0.001	1.923 (1.742-2.122)	<0.001
**Surgical divisions**				
Cardiovascular surgical division	Reference		Reference	
Neurosurgical division	1.500 (1.280-1.758)	<0.001	1.656 (1.400-1.959)	<0.001
General-colorectal surgery divisions	4.376 (3.700-5.174)	<0.001	2.317 (1.943-2.762)	<0.001
**Scheduled surgery**	0.520 (0.475-0.569)	<0.001	0.632 (0.574-0.697)	<0.001
**Severity and managements**				
APACHE II score	1.075 (1.068-1.081)	<0.001	1.031 (1.024-1.039)	<0.001
Presence of shock	2.358 (2.108-2.637)	<0.001	1.290 (1.146-1.451)	<0.001
Receiving mechanical ventilation	1.612 (1.451-1.792)	<0.001	1.285 (1.150-1.435)	<0.001
Receiving RRT	4.144 (3.672-4.677)	<0.001	2.118 (1.858-2.413)	<0.001
**Laboratory data**				
WBC, per 10^3/μL increment	1.006 (0.993-1.019)	0.399	1.011 (0.998-1.024)	0.094
Haemoglobin (g/dL), per 1 increment	0.812 (0.787-0.837)	<0.001	0.935 (0.906-0.965)	<0.001
Albumin (g/dL), per 1 increment	0.504 (0.462-0.550)	<0.001	0.999 (0.999-1.000)	0.131
Creatinine (mg/dL), pre 1 increment	2.294 (2.064-2.551)	<0.001	0.729 (0.665-0.799)	<0.001
ALC, per 10^3 /μL decrement	1.288 (1.237-1.34)	<0.001	1.363 (1.215-1.529)	<0.001

Abbreviations: CCI, Charlson comorbidity index; APACHE, acute physiology and chronic health evaluation; RRT, renal replacement therapy; WBC, white blood cell count; ALC, absolute lymphocyte count.
