# Supplementary material for: Early absolute lymphocyte count was associated with one-year mortality in critically ill surgical patients: A propensity score-matching and weighting study
Source: PLoS One. 2024 May 30;19(5):e0304627. doi: 10.1371/journal.pone.0304627 (PMC11139264; doi:10.1371/journal.pone.0304627)
Supplement: S3 Table — (PDF) [file pone.0304627.s005.pdf]

Supplemental Table 3. Subgroup analysis categorized by surgical types and divisions for the association between absolute lymphocyte count and long-term overall mortality in critically ill surgical patients

	Univariable Analysis		Multivariable Analysis	
	**HR (95% CI)**	**p-value**	**HR (95% CI)**	**p-value**
**Non-emergent surgery**				
**Surgical divisions**				
Cardiovascular surgical division	Reference		Reference	
Neurosurgical division	1.609 (1.349-1.918)	<0.001	1.840 (1.532-2.210)	<0.001
General surgery divisions	4.416 (3.578-5.451)	<0.001	2.338 (1.881-2.905)	<0.001
Colorectal surgery divisions	5.571 (4.504-6.892)	<0.001	2.674 (2.144-3.335)	<0.001
**Emergent surgery**				
**Surgical divisions**				
Cardiovascular surgical division	Reference		Reference	
Neurosurgical division	0.723 (0.497-1.053)	<0.001	1.001 (0.684-1.465)	0.996
General surgery divisions	1.310 (0.829-2.071)	<0.001	0.978 (0.618-1.549)	0.925
Colorectal surgery divisions	2.487 (1.619-3.82)	<0.001	1.658 (1.076-2.556)	0.022
