# Supplementary material for: Early absolute lymphocyte count was associated with one-year mortality in critically ill surgical patients: A propensity score-matching and weighting study
Source: PLoS One. 2024 May 30;19(5):e0304627. doi: 10.1371/journal.pone.0304627 (PMC11139264; doi:10.1371/journal.pone.0304627)
Supplement: Attachment — Submitted filename: Point-by-point reply 2023 1122.docx [file pone.0304627.s007.docx]

**Reviewer #1**

**Q1**. Logic of the study could be expressed better. Hemogram derived inflammatory markers are associated with mortality of intensive care population (Journal of Intensive Care Medicine, 2019, 34.6: 511-513.). On the other hand, lymphocyte or lymphocyte based markers are also associated with mortality (Cirugía y cirujanos, 2022, 90.5: 596-601.). Thus, absolute lymphocyte count could be reasonable to study.

**Reply**

- We thank the reviewer for the thorough reading and the insightful suggestions. We have included the references [Reference 6 and 11] and revised the background as well as the discussion. Please refers to line 42-48 (background) and line 187-189 (discussion).
- Line 42-48
- A low absolute lymphocyte count (ALC) is one of the immune-compromised biomarkers in critically ill patients and has been implicated with vulnerability for secondary infection or infection resulting from opportunistic pathogens in patients who were admitted to the intensive care unit (ICU) [3-6]. Increasing studies have shown the association between ALC and outcomes in critically ill patients, but the majority of studies were conducted in medical or mixed ICUs [7-11]. Few studies have explored the association between low ALC and outcomes in critically ill surgical patients [12-14].
- Line 187-189

One recent study, investigating 5321 patients with coronavirus disease 2019 (COVID-19) infection, also found a lower ALC in deceased patients than those in survived patients (0.9 vs 1.4 k/mm3, p<0.001) [11].

**Q2.** p values of the hazard ratios along with 95% CI could be expressed in results.

**Reply**

- We have added the p values of the HR along with 95% CI in the results. Please refers to line 132-134 and line 151-153.
- Line 132-134

We found that a low ALC was independently associated with increased one-year mortality (adjHR 1.140, 95%CI 1.091–1.192, p<0.001) after adjusting for covariates (Table 2).

- Line 151-153

The adjusted HRs in original, PSM, IPTW, and CBPS populations were 1.497 (95% CI 1.320–1.697, p<0.001), 1.391 (95% CI 1.169–1.654, p<0.001), 1.512 (95% CI 1.310–1.744, p<0.001), 1.511 (95% CI 1.310–1.744, p<0.001), respectively.

**Reviewer #2:**

The manuscript Early absolute lymphocyte count was associated with one-year mortality in critically ill surgical patients: a propensity score-matching and weighting study studied the association between lymphocyte count and mortality of the critically ill surgical patients. There are some points to correct.

**Q1.** Unit of Body mass index must be provided in table 1.

**Reply**

- We thank the reviewer for reminding us of providing the unit of body mass index. We have added the unit (kg/m2) of body mass index in the Table 1.

**Q2.** Similarly, unit of sex must be stated as n(%). Same for other categorical variables.

**Reply**

We have presented the categorical variables as N (%).

**Q3.** Discussion should be improved by discussing lymphocyte or lymphocye including indexs' role in patients required intensive care.

**Reply**

- We have added the discussion with regard to the potential rationale (Line 229-239) and clinical relevance (Line 165-167) of ALC in critically ill patients.
- Line 229-239 (potential rationales)
- Increasing human and animal studies have demonstrated lymphopenia and increased apoptosis in those receiving surgery or anaesthesia [30, 31]. Hotchkiss RS *et al*. compared intestinal tissues obtained from 10 patients with acute traumatic injury to 6 control patients undergoing selective bowel resections and found extensive epithelial damage and lymphocyte apoptosis in the traumatic patients, and the degrees of tissue lymphocyte apoptosis was associated with a decreased circulating lymphocyte count [30]. Yamada *et al*. studied dogs receiving anaesthesia with and without laparotomy and found that lymphopenia was observed in both groups after anaesthesia, with more apoptotic cells in dogs receiving laparotomy than those without laparotomy [31]. These data showed the plausbile mechanisms underlying lymphopenia in surgical patients, and our data further provided clinical evidence of the long-term impact of lymphopenia in critically ill patients.
- Line 165-167 (clinical relevance)

These findings suggest that low week-one ALC, a ready-to-use biomarker in critical care, may serve as a predictor for long-term mortality in critically ill surgical patients.

**Reviewer #3:**

Comments:

I read with great interest the manuscript “Early absolute lymphocyte count was associated with one-year mortality in critically ill surgical patients: a propensity score-matching and weighting study”. In this manuscript the authors claim an association between absolute lymphopenia during the first week of ICU admission and long-term prognosis. This data is interesting, although it has been shown for other ICU populations and the hypothesis this would not be true for surgical patients and the reason why this research is relevant should be better explained in the background.

**Major comments**

**Q1**. The use of the average value of ALC during the first week although pragmatic might introduce some bias in data interpretation. It is likely that during this first week a shift in lymphocyte counts occur. For the ones where more than 1 data point is available what is the variability of the values? If you just use the first ALC or the shift in ALC do you still have the same results?

**Reply**

- We thank the reviewer for this comment and have conducted the analysis using the first level of ALC, and the data were consistent with the analysis using the average during the first week (Supplemental Table 2). Additionally, we have acknowledged that we could not detailly clarify the dynamic change of ALC in this study. Please refer to the supplemental Table 2 and the limitation section (Line 261-264).
- Line 261-264 (limitation section)

Fourth, we used the average level of ALC in this study, and the data were consistent using the first ALC (Supplemental Table 2). More studies are warranted to explore the impact of dynamic change of ALC on long-term mortality in critically ill surgical patients.

| **Supplemental Table 2. Cox proportional hazard regression analysis for the association between the first absolute lymphocyte count and long-term mortality in the critically ill surgical patients** | | | | |
| --- | --- | --- | --- | --- |
|  | **Univariable Analysis** | | **Multivariable Analysis** | |
|  | **HR (95% CI)** | **p-value** | **HR (95% CI)** | **p-value** |
| **Demographic and comorbidity** |  |  |  |  |
| Age, years | 1.027 (1.024-1.030) | <0.001 | 1.009 (1.006-1.013) | <0.001 |
| Sex (male) | 1.289 (1.172-1.418) | <0.001 | 1.150 (1.041-1.270) | 0.006 |
| Body mass index (per 1 kg/m2 increment) | 0.949 (0.938-0.959) | <0.001 | 0.962 (0.952-0.972) | <0.001 |
| CCI ≥ 2 | 2.665 (2.427-2.927) | <0.001 | 1.923 (1.742-2.122) | <0.001 |
| **Surgical divisions** |  |  |  |  |
| Cardiovascular surgical division | Reference |  | Reference |  |
| Neurosurgical division | 1.500 (1.280-1.758) | <0.001 | 1.656 (1.400-1.959) | <0.001 |
| General-colorectal surgery divisions | 4.376 (3.700-5.174) | <0.001 | 2.317 (1.943-2.762) | <0.001 |
| **Scheduled surgery** | 0.520 (0.475-0.569) | <0.001 | 0.632 (0.574-0.697) | <0.001 |
| **Severity and managements** |  |  |  |  |
| APACHE II score | 1.075 (1.068-1.081) | <0.001 | 1.031 (1.024-1.039) | <0.001 |
| Presence of shock | 2.358 (2.108-2.637) | <0.001 | 1.290 (1.146-1.451) | <0.001 |
| Receiving mechanical ventilation | 1.612 (1.451-1.792) | <0.001 | 1.285 (1.150-1.435) | <0.001 |
| Receiving RRT | 4.144 (3.672-4.677) | <0.001 | 2.118 (1.858-2.413) | <0.001 |
| **Laboratory data** |  |  |  |  |
| WBC, per 10^3^/μL increment | 1.006 (0.993-1.019) | 0.399 | 1.011 (0.998-1.024) | 0.094 |
| Haemoglobin (g/dL), per 1 increment | 0.812 (0.787-0.837) | <0.001 | 0.935 (0.906-0.965) | <0.001 |
| Albumin (g/dL), per 1 increment | 0.504 (0.462-0.550) | <0.001 | 0.999 (0.999-1.000) | 0.131 |
| Creatinine (mg/dL), pre 1 increment | 2.294 (2.064-2.551) | <0.001 | 0.729 (0.665-0.799) | <0.001 |
| ALC, per 10^3^ /μL decrement | 1.288 (1.237-1.34) | <0.001 | 1.363 (1.215-1.529) | <0.001 |
| Abbreviations: CCI, Charlson comorbidity index; APACHE, acute physiology and chronic health evaluation; RRT, renal replacement therapy; WBC, white blood cell count; ALC, absolute lymphocyte count. | | | | |

**Q2**. Although this is an important population of surgical population, it is expected to have very different behaviors for the ones with elective surgery versus urgent surgery. This study has a disproportionate high rate of elective surgeries compared to emergent surgeries. This should be interpreted carefully also in the discussion.

**Reply**

- We totally agree with the reviewer that we should interpret this point with caution. We have conducted additional analysis subgroup analysis categorised by surgical types and divisions and found a similar trend among those receiving emergent abdominal surgery (please refers to Q3). We have added a discussion and hedge statement with regard to this crucial point; please refers to line 194-202. Given that we have matched surgical divisions and types of surgery in the propensity score-based analysis, the main claim of the association between ALC and long-term mortality in the study population should not be affected by the surgical division/type.
- Line 194-202

With regards to the role of ALC among subgroups of surgical patients, we found that patients of general-colorectal surgery divisions had a slighter higher mortality than those in the cardiovascular surgical division, implicating the potential role of existing infection in patients receiving abdominal surgery. The stratified analyses found no modification effect of surgical divisions on the association between ALC and long-term mortality (Table 3). We further conducted subgroup analysis categorised by surgical types and divisions and found a similar trend among those receiving emergent abdominal surgery, who were critically ill surgical patients with increased risk for existing infection (Supplemental Table 3). However, we think that more studies are required to clarify the role of ALC in distinct surgical patients

| **Supplemental Table 3. Subgroup analysis categorized by surgical types and divisions for the association between absolute lymphocyte count and long-term overall mortality in critically ill patients** | | | | |
| --- | --- | --- | --- | --- |
|  | **Univariable Analysis** | | **Multivariable Analysis** | |
|  | **HR (95% CI)** | **p-value** | **HR (95% CI)** | **p-value** |
| **Non-emergent surgery** |  |  |  |  |
| **Surgical divisions** |  |  |  |  |
| Cardiovascular surgical division | Reference |  | Reference |  |
| Neurosurgical division | 1.609 (1.349-1.918) | <0.001 | 1.840 (1.532-2.210) | <0.001 |
| General surgery divisions | 4.416 (3.578-5.451) | <0.001 | 2.338 (1.881-2.905) | <0.001 |
| Colorectal surgery divisions | 5.571 (4.504-6.892) | <0.001 | 2.674 (2.144-3.335) | <0.001 |
| **Emergent surgery** |  |  |  |  |
| **Surgical divisions** |  |  |  |  |
| Cardiovascular surgical division | Reference |  | Reference |  |
| Neurosurgical division | 0.723 (0.497-1.053) | <0.001 | 1.001 (0.684-1.465) | 0.996 |
| General surgery divisions | 1.310 (0.829-2.071) | <0.001 | 0.978 (0.618-1.549) | 0.925 |
| Colorectal surgery divisions | 2.487 (1.619-3.82) | <0.001 | 1.658 (1.076-2.556) | 0.022 |

**Q3**. In addition, it is likely that for the ones with urgent colo-rectal surgery, sepsis would be a frequent underlying diagnosis. Do you have data on this? Can sepsis explain partially your results?

**Reply**

- We must thank the reviewer for pointing out this point, and the analysis does show the consistent association between ALC and long-term mortality in patients receiving emergent colo-rectal surgery. To avoid redundancy, please refers to the supplemental Table 3 and the reply to Q2.

**Q4**. Although interesting, it is unexpected that you found a higher association of low lymphocyte counts with a younger age. Do you have a bias towards age for other variables, such as distribution of type of surgery or sepsis? Did you check for interactions between these relevant variables that might explain this finding? I understand your reasoning in the discussion (line 199-206) to explain this finding, but have you also considered the possibility of a relevant bias here? If what you claim is true have you analysed the association between ALC (first week, and probably and frequently first days) and short-term mortality?

**Reply**

- We thank the reviewer for this comment on the result regarding the effect of modification, which was also our concern in the manuscript preparation. The focus of this study is the association between ALC and long-term mortality in critically ill surgical patients, and we have used both Cox regression and propensity-based analyses to demonstrate the association between ALC and long-term mortality. The effect of modification is the additional analysis based on the Cox regression, and we totally agree with the reviewer that we should interpret the finding with caution. We have revised the discussion and added a number of hedge statements regarding this point. Please refers to line 210-221 for the revised discussion with hedge statements. But, this study not only used Cox regression but also applied the propensity score-based analyses to demonstrate the consistent association between ALC and long-term mortality in critically ill surgical patients.
- Line 210-221

In the present study, the analysis of the modification effect showed that the impact of low ALC on mortality tended to be stronger in patients with expected good outcomes, including younger patients and those with fewer comorbidities and lower severities of critical illness (Table 3). We think that the aforementioned finding should be interpreted with caution and postulate that the aforementioned finding suggests that low ALC might possibly reflect the lasting compromised immunity with prolonged impact on the recovery from critical illness in patients with lower severity/comorbidity, whereas mortality among those with high severity/comorbidities is mainly determined by the initial critical illness. Therefore, the mortality impact of early ALC tended to be prominent among those with expected good outcome, and more studies are required to validate this finding. Furthermore, we acknowledge that it is somehow difficult to differentiate the role of ALC in short-term outcome from those in long-term outcome, given that low ALC-relevant effects should be lasting.

**Minor comments**

**Q5**. In flowchart 1 maybe the term mortality is not properly applied as you are ment oning absolute events. Death?

**Reply**

- We thank the reviewer for this suggestion for explicit wording. We have replaced the “mortality” with “death” in the Fig. 1.

**Q6**. In the results section (lines 119-126) it would be useful to refer what you consider the non-surviving group (hospital? 90 days? 1-year?) and be clear about that.

**Reply**

- We thank the reviewer for this suggestion to avoid ambiguity and have revised the sentence by replacing “non-surviving group” with “patients deceased within one year”. (please refers to line 119)
