# Supplementary material for: Early absolute lymphocyte count was associated with one-year mortality in critically ill surgical patients: A propensity score-matching and weighting study
Source: PLoS One. 2024 May 30;19(5):e0304627. doi: 10.1371/journal.pone.0304627 (PMC11139264; doi:10.1371/journal.pone.0304627)
Supplement: Attachment — Submitted filename: Point-by-point reply 2024 0412.docx [file pone.0304627.s008.docx]

**Reviewers' commet**

**Reviewer #1:** PONE-D-23-22982R1 id no article is carefully re-evaluated. Authors' revisions are satisfactory. It is publishable in the journal in the latest form.

**Reply**

- We thank the reviewer for the thorough reading and the insightful suggestions in the process of revision.

**Reviewer #2**: Authors adequately revised the manuscript according to my review recommendations. No other points to suggest further revision. I think it is acceptable for publication.

**Reply**

- We thank the reviewer for the thorough reading and the insightful suggestions in the process of revision.

**## Reviewer #3:**

In this manuscript, the authors claim an association between absolute lymphopenia during the first week of ICU admission and long-term prognosis. This data is interesting, although it has been shown for other ICU populations and the hypothesis this would not be true for surgical patients and the reason why this research is relevant should be better explained in the background.

**Q1.** It is preferable to more explain the importance of the topic for surgical patients in the background section.

**Reply**

- We thank the reviewer for this suggestion that we should elaborate on the research niche, lack of evidence focusing on long-term outcome in critically ill surgical patients, and we have revised the background. Please refers to line 45-58
- Line 45-58
- Increasing studies have shown the association between ALC and outcomes in critically ill patients, but the majority of studies were conducted in medical or mixed ICUs [7-11]. Few studies have explored the association between low ALC and outcomes in critically ill surgical patients [12-14].
- Notably, the aforementioned low ALC-associated immunosuppressed state in acute illness may last after the stabilization of acute illness and contribute to prolonged pathophysiological abnormalities among patients discharged from the ICUs, which have been known as post-intensive care syndrome (PICS) [15, 16]. Additionally, significant progress in critical care medicine over the past twenty years has not only decreased mortality rates in ICUs but also resulted in a steadily growing number of patients being discharged from these units [17]. Increasing studies, including our previous studies, have addressed the long-term outcomes of critically ill patients in medical ICUs [18-20]. However, the role of early low ALC in the long-term outcome among critically ill patients, particularly those who were admitted to surgical ICUs, remains unclear.

**Q2**. In the results section (lines 119-126) it would be useful to refer what you consider the non-surviving group (hospital? 90 days? 1-year?) and be clear about that. The phrase "patients deceased within one year" should not be replaced with the non-survivor group, but rather should be included in the explanation since the term non-survivor group is referenced later.

**Reply**

- We thank the reviewer that we should precisely describe the data, particularly in this study focused on the long-term outcome in critically ill patients. To avoid misunderstanding of the “non-surviving group”, we have replaced “non-surviving group” with “patients deceased within one year” as suggested. Please refers to line 127, 130, and 135-316.

**Q3.** Two supplementary remarks are provided below, which the authors should take into consideration:

**Q3-1.** In all related tables, the term "surgical patients" should be mentioned, for example, table 2 should be modified.

**Reply**

- We thank the reviewer for this suggestion and have revised the manuscript as suggested. Please refers to the revised table 2, supplemental table 3, and the legend of supplemental figure 2.

**Q3-2.** In the title, the term "absolute lymphocyte count" is referenced, whereas, in the background, the study's objective is delineated as investigating the association between week-one ALC and long-term mortality, which appears to need correction.

**Reply**

- We thank the reviewer for this suggestion and have revised the manuscript as suggested. Please refers to line 59.

**Reviewer #4**:

Overall, the paper appears well-structured and comprehensive, covering various aspects of the study from methods to results and discussion. Here are some suggestions for improvement:

**Q1**- Ensure that the language throughout the paper is clear and precise. For example, in the Methods section, you could clarify the term "week-one ALC" to ensure readers understand it refers to the absolute lymphocyte count within the first week of ICU admission.

**Reply**

- We thank the reviewer-4 and reviewer-3 that we should precisely describe the exposure in this study. We have revised the manuscript as suggested. Please refers to line 74, 77, and 78.

**Q2**- The statement regarding ethical approval is clear, but you could briefly mention any specific ethical considerations or guidelines followed during the study, especially concerning patient data and privacy.

**Reply**

- We have added that this study was performed in accordance with the Declaration of Helsinki. Please refers to line 66-68.
- Line 66-68

The study was performed in accordance with the Declaration of Helsinki [21]. The Institutional Review Board of the TCVGH approved the study and waived the informed consent because all of the data were de-identified (Approval No. CE23045C).

**Q3**- It's important to provide details about how covariates were selected and why they were considered relevant. This could enhance the understanding of the readers regarding the adjustments made in the analysis.

**Reply**

- We thank the reviewer for this suggestion and have added details on the selection of variables in the Cox regression. Please refers to line 96-99.
- Line 96-99

Variables were included in the multivariable model if the associated univariable p value was < 0.20 and the variance inflation factor was < 10 [23]. We further included white blood cell count, given that white blood cell count appears to be a known prognosis-relevant variable in critically ill patients [24]

**Q4**- While you've described the statistical methods used, consider providing a bit more context on why each method was chosen and how it contributes to the analysis.

**Reply**

- We are grateful for this suggestion to highlight that we employed both propensity score-matching and -weighting methods in this study and found a robust association between week-one absolute lymphocyte count and one-year mortality in critically ill surgical patients. The revised details with regard to the statistical methods are now added in line 102-106 (methodology) and line 254-262 (discussion section).
- Line 102-106

We further employed not only propensity score-matching (PSM) but also propensity score-weighting methods, including the inverse probability of treatment weighting (IPTW) and the covariate balancing propensity score (CBPS), to confirm the relationship between the low ALC and high one-year mortality among the enrolled critically ill surgical patients [25-27].

- Line 254-262

PSM has been increasingly applied in the past two decades to mitigate confounding effects on research outcomes; however, selecting a subset of individuals from case and control groups based on similar propensity scores raises concerns about the generalizability of the findings [40]. IPTW addresses this issue by using the inverse of the propensity score as a weighting factor for each participant without excluding anyone, thus reducing concerns about generalizability [41]. Nonetheless, IPTW can still be problematic due to the impact of individuals with extreme weights, and CBPS techniques, which include propensity scores as covariates, are less likely to be influenced by individuals with non-overlapping propensity scores [42].

**Q5**- Results Interpretation: Ensure that the results are interpreted accurately and avoid overgeneralization. For instance, when discussing mortality rates, it may be helpful to provide context by comparing them to similar studies or national averages.

**Reply**

- We thank the reviewer for pointing out that we should further elaborate the generalization issue of our findings. The revised discussion on this issue has been added in line 183-188.
- Line 183-188

Few studies focused on the long-term outcome in critically ill surgical patients. Ou *et al.* conducted a population-based study with 1857 critically ill surgical patients with post-operative sepsis and reported that the one-year post-hospital survival rate among critically ill surgical patients with post-operative sepsis was 86.3% (1606/18578) [31]. The aforementioned data were similar to our data in that the one-year post-hospital survival rate was 85.6% (6107/7135) among patients discharged from the surgical ICUs in this study (Table 1).

**Q6**- Discussion: The discussion is insightful, but you could expand on potential implications of the findings for clinical practice or future research directions. Additionally, consider discussing any limitations in more detail and how they may have influenced the results.

**Reply**

- We have revised the conclusion section, please refers to the reply of Q7. Additionally, we have expanded the discussion regarding the limitations of this study. Please refers to line 272-278 in the limitation section.
- Line 272-278

Third, we could not assess the long-term outcomes other than mortality, such as functional status and quality of life. Future studies should aim to incorporate a broader range of outcomes to provide a more holistic view of patient recovery from critical illness. Fourth, we used the average level of ALC in this study, and the findings were consistent using the first ALC (Supplemental Table 2). More studies are warranted to determine the effect of dynamic change of ALC on long-term mortality in critically ill surgical patients.

**Q7**- Conclusion: The conclusion summarizes the findings well, but you could **r**eiterate the key implications of the study and emphasize the need for further research to validate the findings and explore underlying mechanisms.

**Reply**

- We thank the reviewer for the suggestion that we should point out the practical implication and future work in the conclusion section. Please refers to line 279-286
- Line 279-286

In conclusion, the consistent association between low ALC levels and increased one-year mortality, as established through methodologies like Cox regression and propensity score analyses, underscores the crucial role of ALC in critically ill surgical patients. The identification of week-one ALC as an early determinant of long-term outcomes highlights the potential use of week-one ALC as a valuable tool for risk stratification, which may enable more personalized, timely interventions in critically ill surgical patients. Further research is necessary to confirm our findings and to elucidate the biological mechanisms, thereby improving patient management strategies in critical care.

**Reviewer #5:** The manuscript is highly valuable and well-written. The reviewers' comments were correctly and fairly fully applied, except for a few that have received little attention. It is anticipated that applying these suggestions will enhance the article.

**Reply**

- We thank the reviewer for the thorough reading and the insightful suggestions in the revision process.

**Reviewer #6:** The article is well-written and structured, providing an in-depth analysis of the association between week-one ALC and one-year mortality in critically ill surgical patients. The authors have used appropriate statistical methodology to evaluate the desired association and have performed sensitivity analyses with different analytical strategies. Moreover, the authors have addressed all the concerns of the reviewers properly and comprehensively.

**Reply**

- We thank the reviewer for the thorough reading and the insightful suggestions in the revision process.
